# Inhibition of cell growth by BrMC through inactivation of Akt in HER-2/neu-overexpressing breast cancer cells

**DOI:** 10.3892/ol.2014.1889

**Published:** 2014-02-18

**Authors:** XIAO-ZHENG CAO, HONG-LIN XIANG, MEI-FANG QUAN, LI-HUA HE

**Affiliations:** Medical College, Hunan Normal University, Changsha, Hunan 410013, P.R. China

**Keywords:** breast cancer, chrysin, BrMC, HER-2/neu, Akt

## Abstract

We previously reported that chrysin (ChR) and its analogs induced cell cycle arrest and apoptosis in human estrogen receptor-positive/-negative breast cancer cells. However, it was unknown whether 8-bromo-7-methoxychrysin (BrMC), a novel synthetic ChR analog, inhibited the cell growth of human epidermal growth factor receptor 2 (HER-2)/neu-overexpressing breast cancers. In the present study, it was demonstrated that BrMC preferentially inhibited the cell viability of HER-2/neu-overexpressing MDA-MB-453 and BT-474 cells. Western blot analysis revealed that HER-2/neu expression and tyrosine phosphorylation were inhibited by BrMC in a concentration-dependent manner; whereas the proteasome inhibitor, MG-132, significantly prevented BrMC-induced HER-2/neu depletion and cell death in MDA-MB-453 cells. This directly indicated that BrMC-induced HER-2/neu depletion and cell growth inhibition was mediated by a proteasomal pathway. BrMC significantly downregulated the expression of cyclin D1, cyclin E and CDK4, followed by the suppression of protein kinase B phosphorylation and downstream effectors, GSK-3β and β-catenin. A colony formation assay also confirmed the growth-inhibitory effects of BrMC. Thus, these findings clearly demonstrate the anticancer activity of BrMC against human HER-2/neu-overexpressing breast cancer cells. Thus, these findings clearly demonstrate the anticancer activity of BrMC against human HER 2/neu-overexpressing breast cancer cells, and highlight BrMC as a promising candidate for breast cancer therapy.

## Introduction

Breast cancer is the most common type of cancer among females, and is the second leading cause of cancer-related mortality worldwide (1). Current treatment for estrogen-receptor (ER)-positive tumors (>60% of breast cancers) includes surgery, whereby gross tumors are removed, which is followed by treatment with drugs. Drug treatments include aromatase inhibitors and antiestrogens, such as tamoxifen, which target the hormone dependence that is demonstrated by these tumors (2). However, such drugs have little efficacy against certain types of breast cancer cells, including human epidermal growth factor receptor 2 (HER-2)/neu-overexpressing breast cancers. The HER-2/neu oncogene is the second member of the HER (also known as ErbB) family (3). More than 30% of breast cancers were identified to exhibit HER-2/neu overexpression, which is considered a predictive marker of resistance to tamoxifen therapy. Aberrant activation of the HER-2 receptor is closely associated with increased metastatic potential and resistance to chemotherapeutic agents (4,5). Activation of receptor tyrosine kinases, such as phosphatidylinositide 3-kinase (PI3K) and protein kinase B (Akt), which have an intrinsic ability to phosphorylate tyrosine residues in their cytoplasmic domains, results in the activation of nuclear transcription factors that induce cell growth and inhibit apoptosis (4). Therefore, inhibition of HER-2/neu has become an important therapeutic target for human breast cancers.

Chrysin (5,7-dihydroxyflavone, ChR) is a naturally occurring, biologically active flavone that has been demonstrated to inhibit cell proliferation and induce apoptotic cell death in a variety of cancer cells, including breast cancer (6–8). Due to poor oral bioavailability, ChR may not be successfully used as a dietary flavonoid for cancer chemotherapeutics (9). We previously demonstrated that the effect of 8-bromo-7-methoxychrysin (BrMC) on the inhibition of proliferation and induction of apoptosis in a colon cancer cell line, HT-29, and a gastric cancer cell line, SGC-7901, was stronger than that of ChR (10). BrMC also exhibited effective inhibition of proliferation and induction of apoptosis in colon, gastric and liver cancer cells (11–13). However, whether BrMC inhibits the cell growth of HER-2/neu-overexpressing breast cancers has not yet been determined.

In the present study, the effectiveness of BrMC against two human breast cancer cell lines exhibiting high levels of HER-2/neu expression was investigated.

## Materials and methods

### Cell culture and reagents

The human breast cancer cell lines, MDA-MB-453 and BT-474 (which endogenously overexpress the HER-2/neu oncogenes) and MCF-7 (low HER-2 expression), as well as the human breast cell line, MCF-10A (low HER-2 expression), were used in this study. The cell lines were obtained from the Cell Bank of the Chinese Academy of Sciences (Shanghai, China), and cells were grown in Dulbecco’s Modified Eagle’s medium (DMEM), with F-12 nutrient mixture, supplemented with 10% heat-inactivated fetal bovine serum (FBS), 2 mM glutamine, and 1% penicillin-streptomycin-neomycin (all purchased from Gibco-BRL, Grand Island, NY, USA) at 37°C in a humidified incubator with 5% CO_2_. BrMC was synthesized as described previously (10). Cultures were harvested and monitored for changes in cell number by counting cell suspensions using a hemocytometer (Model 1280, Shanghai Qiujing Biochemical Reagent and Instrument Co., Ltd., Shanghai, China) with a phase-contrast microscope (BX41, Olympus Optical Co., Ltd., Tokyo, Japan).

### Cell viability assay

Cells were seeded in a 96-well plate at a density of 0.5×10^4^ cells/well and treated with serum-free medium (Gibco-BRL) for 24 h, followed by treatment with various concentrations of experimental agents (Akt inhibitor LY294002: Calbiochem, La Jolla, CA, USA; proteasome inhibitor MG132: Bio Vision, Inc., Mountain View, CA, USA), which were added to each well and cultured for 24 h, followed by incubation with media containing 0.5 mg/ml 3-(4,5-dimethylthiazol-2-yl)-2,5-diphenyltetrazolium bromide (Sigma-Aldrich, St. Louis, MO, USA) for 4 h. The supernatant was removed following centrifugation at 1000 × g for 5 min. Finally, 100 μl dimethyl sulfoxide (Amresco Company, Solon, OH, USA) was added and the absorbance at the 570-nm wavelength (A_570_) was measured using an enzyme-labeling instrument (ELX-800; Bio-Tek Instruments, Inc., Shanghai, China). Cell viability was calculated as follows: Cell viability (%) = (A_570_ of treated cells/A_570_ of untreated cells) ×100.

### Western blot analysis

MDA-MB-453 or BT-474 cells (1.5×10^6^ cells/10-cm dish) were incubated with various concentrations of BrMC for 24 h. After incubation, the cells were washed once in phosphate-buffered saline (PBS), detached, pooled and centrifuged at 1500 × g for 5 min. The cell pellets were subsequently suspended in 100 μl lysis buffer (Sigma-Aldrich; 10 mM Tris-HCl, pH 8.0; 320 mM sucrose; 1% Triton X-100; 5 mM ethylenediaminetetraacetic acid; 2 mM dithiothreitol; and 1 mM phenylmethylsulfonyl fluoride). The suspensions were kept on ice for 20 min and centrifuged at 15000 × g for 30 min at 4°C. Total protein content was determined with the Bio-Rad protein assay reagent (Bio-Rad, Hercules, CA, USA) using bovine serum albumen (Gibco-BRL) as a standard. Protein extracts were reconstituted in sample buffer [Sigma-Aldrich; 62 mM Tris-HCl, 2% sodium dodecyl sulfate (SDS), 10% glycerol, 5% β-mercaptoethanol], and the mixture was boiled at 97°C for 5 min. Equal amounts (50 μg) of denatured protein samples were loaded into each lane, separated by SDS-polyacrylamide gel electrophoresis on an 8–15% polyacrylamide gradient gel and transferred onto polyvinylidene difluoride membranes overnight. The membranes were blocked with 5% non-fat dried milk in PBS containing 1% Tween-20 (Sigma-Aldrich) for 1 h at room temperature, and subsequently incubated with primary antibodies [rabbit polyclonal antibodies against cyclinE, p27^KIP^, p21^CIP^, CDK1, CDK2, and β-actin were purchased from Santa Cruz Biotechnology, Inc. (Santa Cruz, CA, USA). Mouse monoclonal antibodies against HER 2/neu (p185), HER/neu, p-PI3K, PI3K, p-Akt, Akt, β-catenin, p-GSK-3β, GSK-3β, cyclin D1 and CDK4 were obtained from Cell Signaling Technology, Inc. (Danvers, MA, USA)]. for 2 h and either horseradish peroxidase-conjugated goat anti-rabbit or anti-mouse antibodies overnight. The signal intensity was then measured using a chemiluminescent detection system (Pierce Biotechnology, Inc., Rockford, IL, USA).

### Colony formation assay

Anchorage-independent growth was determined by colony formation in soft agar (14). The assay was performed in six-well plates (1×10^4^ cells/well) with a base layer containing 0.5% agar in DMEM containing 10% FBS, 1 mM glutamine, 100 units penicillin and 100 μg/ml streptomycin. This layer was overlaid with a second layer of 1 ml 0.3% agar (in DMEM containing 10% FBS, 1 mM glutamine, 100 units of penicillin and 100 μg/ml of streptomycin) with a suspension of 1×10^4^ cells/well. Fresh medium with either BrMC or ChR was then added to the plates every 72 h. The plates were incubated at 37°C for 3 weeks, and the tumor colonies were then analyzed microscopically. Colonies with a diameter >0.2 mm were counted.

### Statistical analysis

The results are presented as the mean ± standard deviation. All study data were analyzed using analysis of variance followed by Dunnett’s test for pairwise comparison. An asterisk indicates that the experimental values are significantly different from those of the control (^*^P<0.05).

## Results

### BrMC preferentially inhibits cell viability of HER-2/neu-overexpressing breast cancer cells

**Figure 1:**
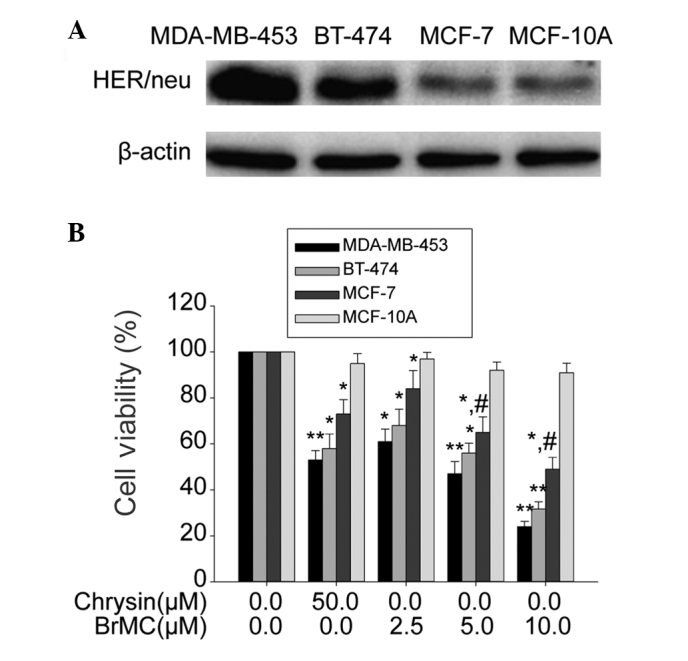
Effects of BrMC on the HER-2/neu expression and proliferation of breast cancer lines (MDA-MB-453, BT-474, MCF-7) and the human breast cell line (MCF-10A). (A) Western blot analysis of the expression of HER-2/neu protein, where β-actin was used as the loading control. (B) After incubation with different concentrations of BrMC (0–10 μM) or chrysin (50 μM) for 24 h, the cell viability was examined by 3-(4,5-dimethylthiazol-2-yl)-2,5-diphenyl tetrazolium bromide assay. Data are shown as the means ± SD, n=3. ^*^P<0.05, compared with respective controls; ^**^P<0.01, compared with respective controls; and ^#^P<0.05, compared with the other cell lines treated with the same concentration of BrMC. BrMC,8-bromo-7-methoxychrysin; HER-2, human epidermal growth factor receptor 2.

A previous study indicated that ChR and its analog (NOC) preferentially inhibited growth in human HER-2/neu-overexpression breast cancer cells (15); therefore, in the present study the effects of BrMC on the growth of three breast cancer lines (MDA-MB-453, BT-474 and MCF-7) and the immortalized noncancerous MCF-10A breast cell line were investigated. The three breast cancer cell lines examined were selected due to their varying levels of HER-2/neu expression. In the present study, it was demonstrated that MDA-MB-453 and BT-474 cell lines harbor HER-2/neu overexpression, and MCF-7 and MCF-10A harbor low expression of HER-2/neu (Fig. 1A). BrMC inhibited cell viability in a dose-dependent manner (Fig. 1B); MDA-MB-453 and BT-474 cell lines were most sensitive, the MCF-7 cell line was moderately sensitive and the MCF-10A cell line was least sensitive to BrMC. These results suggest that BrMC preferentially suppresses the viability of HER-2/neu-overexpressing cell lines, MDA-MB-453 and BT-474.

### BrMC inhibits HER-2/neu protein expression through the regulation of proteasomal activity

**Figure 2:**
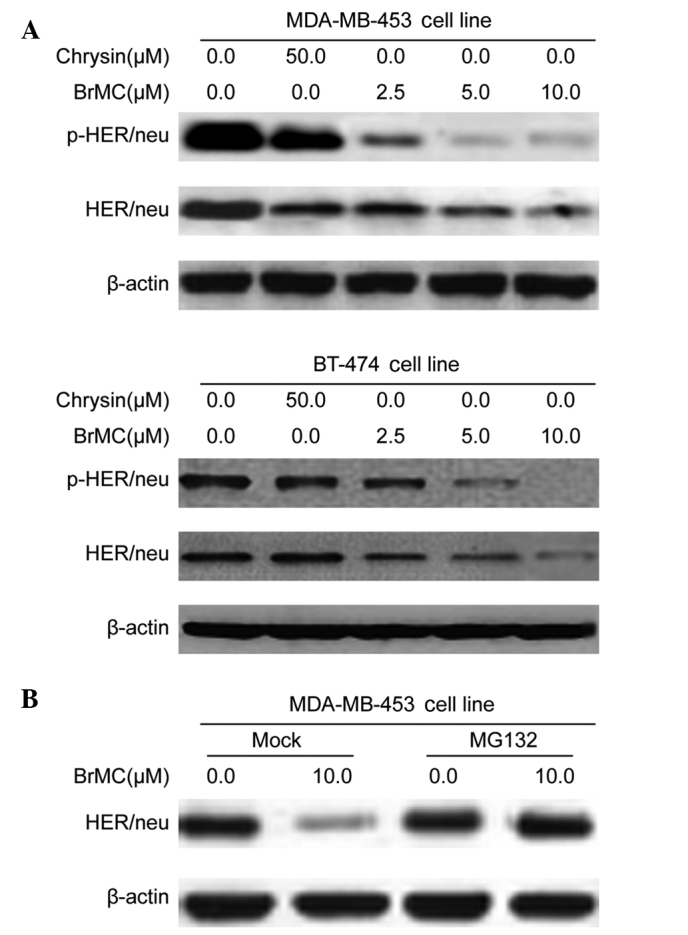
Inhibitory effects of BrMC on HER-2/neu protein phosphorylation and expression in HER-2/neu-overexpressing human breast cancer cell lines. (A) MDA-MB-453 or BT-474 cells were incubated with or without BrMC (0–10 μM) or chrysin (50 μM ) for 24 h. Western blotting was performed to measure levels of the HER-2/neu protein and phosphorylation. β-actin was used as the loading control. (B) MDA-MB-453 cells were pretreated with MG132 (5 μmol/l) for 30 min followed by BrMC (10 μmol/l) for 24 h. Western blotting was performed to measure levels of the HER-2/neu protein. β-actin was used as the loading control. BrMC, 8-bromo-7-methoxychrysin; HER-2, human epidermal growth factor receptor 2.

Activation of the HER-2/neu network leads to autophosphorylation of the receptor’s C-terminal tyrosine and, subsequently, recruitment of cytoplasmic signal transducers to these sites, which regulates cellular processes, such as proliferation, inhibition of apoptosis and transformation (4). Therefore, the present study investigated whether BrMC treatment could reduce such basal HER/neu phosphorylation. MDA-MB-453 and BT-474 human breast cancer cells were treated with BrMC for 24 h. The total cell lysates were isolated and analyzed by western blot analysis using HER-2/neu and phosphotyrosine-specific HER-2/neu antibodies. Fig. 2A shows that treatment of MDA-MB-453 and BT-474 cells with BrMC (at 2.5, 5.0 and 10.0 μM) and ChR (50 μM) for 24 h resulted in a substantial decrease in HER-2/neu tyrosine phosphorylation. BrMC and ChR treatment similarly reduced basal HER-2/neu levels in both cell lines (Fig. 2A). Overall, these findings indicate that BrMC reduced the basal phosphorylation and constitutive activation of HER-2/neu receptors in HER-2/neu-overexpressing breast cancer cells.

To examine the role of proteolysis in BrMC-mediated HER-2/neu downregulation, MG132, the proteasome inhibitor was used. In the absence of MG132, BrMC treatment significantly reduced the HER-2/neu levels (Fig. 2B). Cotreatment with MG-132 resulted in accumulation of HER-2/neu protein in MDA-MB-453 cells (Fig. 2B). These data suggest that proteasomal activity was critically involved in BrMC-induced HER-2/neu degradation in MDA-MB-453 cells.

### BrMC inhibits the activation of Akt in HER-2/neu-overexpressing breast cancer cells

**Figure 3:**
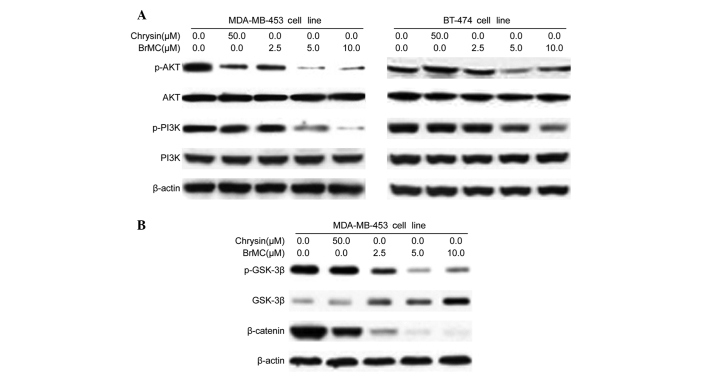
BrMC treatment suppresses the phosphorylation of PI3K/Akt and GSK-3β/β-catenin in HER-2/neu-overexpressing breast cancer cell lines. (A) MDA-MB-453 or BT-474 cells were incubated with or without BrMC (0–10 μM) or ChR (50 μM) for 24 h. Western blotting was performed to measure levels of PI3K and Akt protein and phosphorylation. β-actin was used as the loading control. (B) MDA-MB-453 cells were incubated with or without BrMC (0–10 μmol/l) or ChR (50 μmol/l) for 24 h. Western blotting was performed to measure levels of GSK-3β and β-catenin protein, as well as GSK-3β phosphorylation. β-actin was used as the loading control. BrMC, 8-bromo-7-methoxychrysin; ChR, chrysin; PI3K, phosphatidylinositide 3-kinase; Akt, protein kinase B; HER-2, human epidermal growth factor receptor 2.

A key mechanism by which HER-2/neu overexpression stimulates tumor cell growth and renders cells chemoresistant involves the HER-2/neu receptor. This mechanism also involves Akt kinase; human breast cancer cells with overexpression and amplification of HER-2/neu have been shown to increase Akt kinase activity (4). The involvement of HER-2/neu in the activation of the PI3K/Akt signaling pathway in MDA-MB-453 and BT-474 cell lines was investigated. BrMC and ChR treatment significantly inhibited the phosphorylation of Akt in MDA-MB-453 HER-2/neu-overexpressing breast cancer cells in a dose-dependent manner (Fig. 3A). In addition, it was observed that BrMC treatment significantly inhibited the expression of the Akt upstream kinase, PI3K; in MDA-MB-453 and BT-474 cells; however, this effect did not occur with ChR (Fig. 3A). These data established that BrMC-induced HER-2/neu depletion and cell growth inhibition may be mediated by the inactivation of PI3K/Akt activity in HER-2/neu-overexpressing breast cancer cells.

When Akt is active, a number of substrates are activated that are involved in apoptosis, cell cycle regulation and protein synthesis (4). Akt may potentially regulate cell cycle progression by phosphorylating and inactivating GSK-3β, thereby stabilizing nuclear translocation of β-catenin and increasing cyclin D1 and Cdk4 transcription (16). In BrMC-treated MDA-MD-453 cells, phosphorylated GSK-3β levels decreased substantially, whereas total GSK-3β levels increased (Fig. 3B). This observation suggests that the treatment of cells with BrMC augmented the activity of GSK-3β. Levels of β-catenin, a key component of the Wnt signaling pathway, which is degraded via polyubiquitination upon phosphorylation by GSK-3β, decreased substantially following BrMC treatment (Fig. 3B). In conclusion, our data demonstrated that BrMC may inhibit cell proliferation by suppressing GSK-3β and the β-catenin pathway in HER-2/neu-overexpressing breast cancer cells.

### BrMC regulates cell cycle regulatory proteins in HER-2/neu-overexpressing breast cancer cells

**Figure 4:**
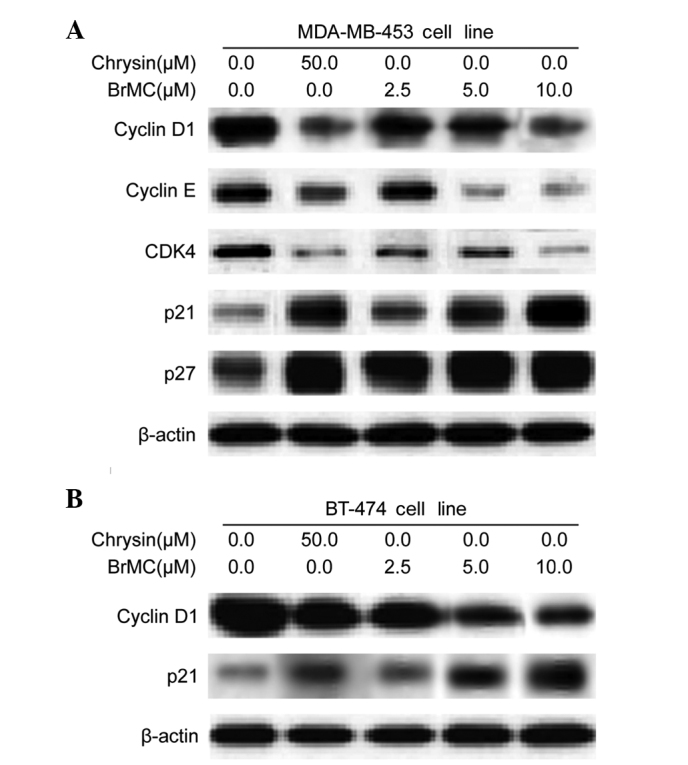
BrMC alters cell cycle regulatory proteins in HER-2/neu-overexpressing breast cancer cells. (A) MDA-MB-453 cells were incubated with BrMC (0–10 μM) or ChR (50 μM) for 24 h. Cyclin D1, cyclin E, p21CIP, p27KIP, Cdk4 and β-actin protein levels were analyzed by western blotting. (B) BT-474 cells were treated with BrMC (0–10 μM) or ChR (50 μM) for 24 h. Cyclin D1, p21CIP, and β-actin protein levels were analyzed by western blotting. BrMC, 8-bromo-7-methoxychrysin; ChR, chrysin; HER-2, human epidermal growth factor receptor 2.

To examine the molecular mechanism(s) and underlying changes in cell cycle patterns caused by BrMC treatment, the effects of various cyclins and Cdks involved in cell cycle regulation were investigated, in MDA-MB-453 cells. BrMC treatment for 24 h caused a dose-dependent reduction of cyclin D1 and cyclin E expression in HER-2/neu-overexpressing MDA-MB-453 cells (Fig. 4A). Cyclin D1 serves as the regulatory subunit of Cdk4 and contributes to its stability. Therefore, the effects of BrMC on Cdk expression were assessed; treatment of MDA-MB-453 cells with BrMC resulted in a dose-dependent decrease in Cdk4 expression (Fig. 4A). However, there was no change in the Cdk1 and Cdk2 protein levels (data not shown). These results imply that BrMC inhibits cell cycle progression by reducing the levels of cyclin D1, cyclin E and Cdk4 in MDA-MB-453 cells. In addition, Akt may contribute to the induction of cell cycle progression by regulating the Cdk inhibitors, p27^KIP^ and p21^CIP^ (17). Both p27^KIP^ and p21^CIP^ protein levels dose-dependently increased in response to BrMC treatment (Fig. 4A). A similar pattern of results was also observed in BT-474 cells. BrMC treatment caused downregulation of cyclin D1 and upregulation of p21^CIP^ expression in a dose-dependent manner in BT-474 cells (Fig. 4B).

### BrMC inhibits anchorage-independent growth of HER-2/neu-overexpressing breast cancer cells

**Figure 5:**
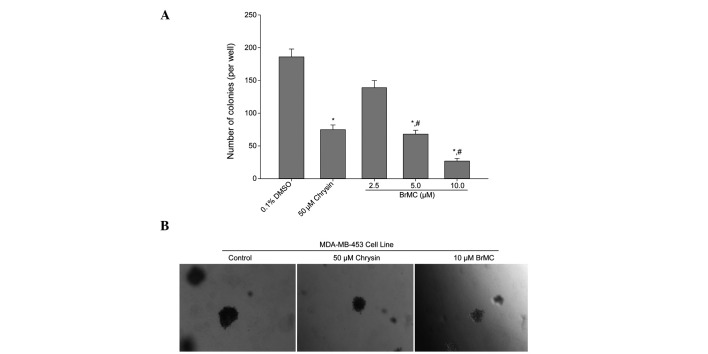
BrMC inhibits anchorage-independent growth of MDA-MB-453 cells. (A) MDA-MB-453 cells were assayed for their ability to form colonies in soft agar. Cells were seeded onto 6-cm dishes in culture medium containing 0.3% low-melting agarose over a 0.6 % agarose layer in the presence of BrMC (0–10 μM) or chrysin (50 μM) and incubated for 2 weeks at 37°C. The percentage colony formation was calculated by defining the number of colonies in the absence of BrMC as 100%. Data are shown as the means ± SD, n=3. ^*^P<0.05, compared with the control; and ^#^P<0.05, compared with 2.5 μmol/l BrMC. (B) The photomicrographs shown here are from one representative experiment repeated twice with similar results. BrMC, 8-bromo-7-methoxychrysin.

The effect of BrMC upon anchorage-independent colony growth in soft agar was determined. Anchorage-independent growth is a property of transformed and tumor cells, and is closely correlated with tumorigenesis *in vivo*. Colony formation of MDA-MB-453 cells, which are known to overexpress HER-2/neu, was significantly (P<0.05) suppressed by BrMC treatment as compared with that in the control group (Fig. 5A). Reductions in colony number were accompanied by a reduction in colony size in MDA-MB-453 cells (Fig. 5B). Therefore, the data indicate that BrMC treatment suppressed the transformation ability of HER-2/neu-overexpressing breast cancer cells.

## Discussion

Previous studies have shown that 5,7-dihydroxy-8-nitrochrysin, a novel synthetic ChR analog, preferentially suppresses the viability of the MDA-MB-453 human breast cancer cell line (ER-negative, HER-2-overexpressing) and moderately suppresses the viability of the MCF-7 cell line (ER-positive, HER-2-low), but has little effect on the immortalized noncancerous HBL-100 breast cell line (ER-positive, HER-2-low) (15). This finding indicated that the novel synthetic ChR analog was differentially cytotoxic towards different breast cancer cell lines without exerting harmful effects on normal cells at higher concentrations.

In the present study, BrMC (another synthetic ChR analog) was observed to mediate inhibition of cell proliferation in HER-2/neu-overexpressing human breast cancer cells. It was demonstrated that BrMC treatment efficiently inhibited the cell viability of MDA-MB-453 and BT-474 cells with an IC_50_ value of 6.2 and 7.4 μmol/l, respectively. It was also revealed that exposure of the HER-2/neu-overexpressing breast cancer cells to BrMC resulted in HER-2/neu depletion and downregulation of PI3K/Akt signaling cascades. Thus, such data indicate that BrMC may be used as a possible chemopreventive or chemotherapeutic agent against human breast cancers.

Overexpression of human epidermal growth factor receptor-2 (HER-2/neu) has been frequently observed in breast cancer cells and is known to indicate a poor clinical prognosis. Therefore, drugs that reduce HER-2/neu activity may be a potential target for breast cancer therapy. Depletion of HER-2/neu in HER-2/neu-overexpressing human breast cancer cells has been demonstrated to arrest cell proliferation (4). Trastuzumab (Herceptin), a humanized antibody that targets the extracellular domain of HER-2/neu, has become a commercialized medicine for the treatment of HER-2/neu-overexpressing early-stage and metastatic breast cancers. However, when used as a single agent, trastuzumab is beneficial in only 15–30% of HER-2/neu breast cancer patients, which can be significantly increased to 50–80% by the addition of chemotherapeutic agents (18). In the current study, observations showed that BrMC treatment effectively downregulates HER-2/neu protein expression in HER-2/neu-overexpressing MDA-MB-453 and BT-474 human breast cancer cells. It has been previously reported that 5,7-dihydroxy-8-nitrochrysin blocks HER-2/neu expression by inhibiting the phosphorylation of Akt in HER-2/neu-overexpressing MDA-MB-453 breast cancer cells (5). ChR inhibits the tyrosine kinase activity of HER-2/neu and induces HER-2/neu degradation by the proteasome when inhibition of protein degradation by MG-132 leads to the accumulation of the NP-40-insoluble form of HER-2/neu. BrMC-induced growth inhibition increases the susceptibility of HER-2/neu-overexpressing cancer cells (5). These data indicate that BrMC may be a promising anticancer agent for human breast cancers.

Akt kinase and its downstream transcription factors have been studied in detail to determine their role in cell proliferation, survival, cell cycle control and other cellular functions (19). In numerous cell types, PI3K/Akt induces survival in response to a variety of stimuli, including growth factor withdrawal and loss of cell adhesion (20). BrMC was found to have an inhibitory effect on the steady-state levels of total PI3K protein in the present study. Additionally, the phosphorylation of its downstream effector Akt, was inhibited, indicating that the disruption of Akt signaling/Akt inactivation plays a functional role in BrMC-mediated cytotoxicity in HER-2/neu-overexpressing breast cancer cells. The present data also suggested that BrMC-mediated inhibition of cyclin D1 is directly proportional to the suppression of HER-2/neu and PI3K/Akt in human breast cancer cells. Overall, these results suggest that HER-2/neu may regulate cellular cyclin D1 via the PI3K/Akt pathway, implying that PI3K/Akt signaling predominantly contributes to cell cycle progression.

In the present study, it was also demonstrated that BrMC treatment downregulates β-catenin expression through upregulation of its negative regulator, GSK-3β. The BrMC-induced increase in GSK-3β may contribute to its effects on Wnt/β-catenin pathway inhibition. Akt kinase has been shown to phosphorylate several key substrates that regulate protein translation (21). In addition, the phosphorylation of its substrate, GSK-3β, as well as nuclear β-catenin stabilization and increased cyclin D1 transcription have all been demonstrated in MDA-MB-453 cells (4). The Wnt/β-catenin signaling pathway has been shown to play an important role in the regulation of cyclin D1, which is crucial in cell cycle regulation and progression in a variety of tumor cells (16). Recent studies clearly described that the interactions between HER-2/neu and cyclin D1 appear to have therapeutic relevance as several phytochemical or synthetic drugs have been demonstrated to reduce cyclin D1 expression through the inhibition of HER-2/neu, and the anti-HER-2/neu monoclonal antibody trastuzumab (Herceptin) reduces cyclin D1 protein levels in human breast cancer cells (22,23). In addition, the present study results demonstrated that BrMC treatment significantly inhibited MDA-M-453 proliferation, which was associated with the suppression of GSK-3β and β-catenin expression and a decrease in expression of their transcriptional targets, including cyclin D1 and Cdk4.

Anchorage-independent growth is a characteristic of numerous types of tumor cells, which distinguishes them from their normal counterparts (24). Anchorage-dependent growth requires integrin-mediated signaling, which is generated by cellular contact with extracellular matrix ligands (25). Normal cells, particularly epithelial cells, undergo apoptosis if they become detached from their underlying or pericellular matrices, in a process termed anoikis (24). By contrast, a number of tumor and transformed cells have escaped this requirement for survival and growth. Moreover, the ability of HER-2/neu-overexpressing breast cancer cells to grow in an anchorage-independent manner has been linked to elevation of the PI3K/Akt cell survival pathway (26). In the current study, we found that BrMC decreased MDA-MB-453 cell proliferation and markedly reduced their capacity to form colonies in soft agar. The loss of anchorage-independent growth of HER-2/neu-overexpressing breast cancer cells treated with BrMC indicates that these cells may have reverted to a less transformed phenotype. This inhibition may also be mediated by the reduction of PI3K/Akt activation.

We hypothesized that BrMC induced cellular effects, resulting from loss of HER-2/neu expression, which may cause subsequent inactivation of PI3K and Akt in cells that are dependent on this pathway for cell proliferation and inhibition of apoptosis. Results from this study also highlight the importance of HER-2/neu or PI3K/Akt components, including GSK-3β, β-catenin, cyclin D1 and p21^WAF1,^ which may serve as future targets for the development of therapeutic strategies against HER-2/neu-overexpressing breast cancer. The inhibition of cell growth in HER-2/neu-overexpressing breast cancer cells following BrMC administration provides a new strategy for breast cancer therapy. However, *in vivo* studies are required to confirm the pharmacological efficacy and safety of BrMC.
